# In dogs, is concurrent laser treatment more effective at treating osteoarthritis compared to NSAIDs alone?

**DOI:** 10.18849/ve.v10i4.719

**Published:** 2025-10-20

**Authors:** Ciar Fitzpatrick+

**Keywords:** ANALGESIA, CANINE, LASER THERAPY, NON-STEROIDAL ANTI-INFLAMMATORY DRUGS, OSTEOARTHRITIS, PAIN MANAGEMENT

## Abstract

**PICO question:**

In dogs with osteoarthritis (OA) is concurrent laser treatment more effective than non-steroidal anti-inflammatories (NSAIDs) in reducing the severity of clinical signs associated with OA?

**Clinical bottom line:**

**Category of research:**

Treatment.

**Number and type of study designs reviewed:**

One randomised controlled trial.

**Strength of evidence:**

Weak.

**Outcomes reported:**

The study showed that laser therapy significantly improved the Helsinki pain score, reduced the NSAID dose and improved the lameness score compared to the control group whose lameness scores did not improve at all.

**Conclusion:**

It is suggested that laser therapy may be effective at reducing clinical signs of osteoarthritis (OA) and therefore reducing the requirement for higher non-steroidal anti-inflammatory drug (NSAID) doses. However, due to the power of the evidence being weak and limited, further clinical studies would be needed to confirm results and conclude whether laser treatment is superior to NSAID treatment.

## How to apply this evidence in practice

The application of evidence into practice should take into account multiple factors, not limited to: individual clinical expertise, patient’s circumstances and owners’ values, country, location or clinic where you work, the individual case in front of you, the availability of therapies and resources.

Knowledge Summaries are a resource to help reinforce or inform decision-making. They do not override the responsibility or judgement of the practitioner to do what is best for the animal in their care.

## The evidence

The search strategies found one paper that was relevant to the PICO question (Looney et al., 2018). Looney et al. (2018) presented evidence that dogs receiving laser therapy had significant improvements in pain scores, reduced non-steroidal anti-inflammatory drug (NSAID) doses and better lameness scores compared to those treated with NSAIDs alone. This study was a randomised blind controlled trial, with a high rate of patient follow-up, meaning it is a relatively strong source of evidence. However, the study had a small sample size, which may have hindered the ability to randomise groups and apply results to a wider population. For this reason, despite the strength of the type of study, the overall strength of evidence remains weak and further studies are needed.

## Summary of the evidence

**Looney et al. (2018):** A randomized blind placebo-controlled trial investigating the effects of photobiomodulation therapy (PBMT) on canine elbow osteoarthritis **Aim: **To determine the effect of photobiomodulation therapy or sham light therapy on pain and lameness caused by naturally occurring elbow osteoarthritis in dogs.

Population:	Dogs with naturally occurring elbow osteoarthritis (OA), from three hospitals.
Sample size:	20 dogs.
Intervention details:	20 dogs were randomly assigned via a coin toss to: Photobiomodulation (PBMT) group (active laser therapy) (n = 11) Sham light group (placebo treatment) (n = 9). Both groups had twice-weekly treatments conducted by blinded technicians, applied to both elbows. The NSAID type, dose, and frequency was noted at the beginning and six weeks later at the end of the study. After the third week, owners were instructed to half their dogs’ NSAID dose. If the owner perceived that the condition worsened, they could return to the original dosage.
Study design:	Randomised blind placebo-controlled trial.
Outcome Studied:	A blinded clinician assessed the lameness score and pain was assessed by the owners using the Helsinki chronic pain index as a baseline. This was then repeated, by a blinded clinician, six weeks later at the end of the study. Owners were questioned weekly on the mood, vocalisation, activity, appetite, attitude, mobility, focus on area or limb/foot, and ‘normality’ of life to determine whether dosage needed to be changed.
Main Findings (relevant to PICO question):	NSAID dose was able to be reduced by at least 50% in 9/11 dogs in the laser treatment group (P = 0.0003). Lameness and pain scores were improved in the laser treatment group compared to the control group (P = 0.001 and P < 0.05 respectively). Mood and vocal scores did not significantly change between the groups (P = 0.20 and P = 0.35 respectively).
Limitations:	The small sample size hindered randomisation. The NSAIDs prescibed to participants were not standardised. Subjective measures were used to determine whether a reduced dose should be maintained. The length of the study was short: only six weeks. Lameness was only assessed at the beginning and six weeks later at the end of the study.

## Appraisal, application and reflection

The search strategy identified one relevant paper for the PICO question (Looney et al., 2018). According to the evidence pyramid, the use of randomised blinded control trials (RCTs) by Looney et al. (2018) provides a high level of evidence (Chen & Chi, 2023). Looney et al. (2018) investigated whether laser therapy could reduce the dose of nonsteroidal anti-inflammatory drugs (NSAIDs) in dogs with elbow osteoarthritis (OA). NSAIDs are the primary treatment choice for OA dogs in the veterinary practice, and while the incidence of side effects is generally low, minimising risks associated with long-term NSAID use remains an important area of investigation (Magni et al., 2021). Non-steroidal anti-inflammatories alleviate OA symptoms by inhibiting cyclooxygenase enzymes, thus reducing the production of prostaglandins and thromboxane A. In contrast, laser therapy uses light waves to penetrate tissue and modulate biological functions, potentially alleviating OA symptoms by promoting healing in affected areas (Looney et al., 2018; Magni et al., 2021).

The diagnostic procedures in Looney et al. (2018) were standardised across three hospitals, and both assessments and treatments were conducted by blinded clinicians. Owners were also blinded to the treatment groups that their dogs had been assigned to. This contributes to a higher level of evidence as it reduces biases from the owners and the clinicians.

Looney et al. (2018) found that the dogs in the laser therapy group were significantly superior to the control group (P = 0.0003) in maintaining a reduced NSAID dose. Additionally, the laser therapy group showed greater improvements in lameness and pain scores (P = 0.001 and P < 0.05, respectively), though mood and vocalisation scores did not differ significantly (P = 0.20 and P = 0.35, respectively). Therefore, this demonstrates that laser therapy can be beneficial as part of a multi-modal approach to treating OA in dogs. Reducing NSAID doses could potentially reduce the associated side effects, such as gastrointestinal issues and renal toxicity (Pye et al., 2022). Multimodal treatment strategies are commonly used in OA management, as a single treatment modality often does not provide sufficient analgesia (Lascelles et al., 2008). However, traditional multimodal management does not include laser treatment; instead, it often includes NSAIDs, analgesics, exercise modifications, dietary changes and physical rehabilitation (Millis, 2021; Thoene et al., 2023). Future research should investigate how laser therapy can be integrated into these multimodal treatment plans and assess its long-term efficacy.

However, it should be noted that the study has several limitations. The small sample size of 20 dogs reduced the statistical power of the study and could impact the reliability of the results, as well as decrease the generalisability. According to Nüesch et al. (2010), studies with a small sample size are more likely to report a larger treatment effect than studies with a larger sample size. Looney et al. (2018) featured a small sample size due to their strict inclusion criteria, which ensured that participants had no underlying conditions and no other orthopaedic diseases, such as stifle OA. This along with the careful selection of participants could account for the positive treatment affect, as suggested by Nüesch et al. (2010). However, the uneven distribution of dogs, nine dogs in the control group and eleven in the treatment group, due to random allocation via coin toss, could have affected the statistical analysis as just one participant could be the difference between a significant and non-significant result in such a small sample. Future studies with a larger sample and a more balanced allocation of participants are necessary to confirm results. Additionally, the inclusion of both unilaterally and bilaterally affected dogs may have influenced results, as the number of clinically healthy limbs could affect the owner's perception of quality of life.

Nonsteroidal anti-inflammatory drug treatment was not standardised throughout the trial, with some participants switching brand of NSAID mid-study. This variability could have influenced the results, though it reflects real-world clinical practice, where NSAIDs are prescribed based on clinician preference. Pye et al. (2022) suggest that there is currently no evidence to favour one NSAID over another, therefore the impact of varying NSAID use in Looney et al. (2018) may be minimal. However, to minimise this potential confounder, future studies should standardise the NSAID treatment protocol.

Lameness assessments by Looney et al. (2018) were conducted only at the beginning and end of the study. Future research could benefit from more frequent assessments to examine how laser therapy and reduced NSAID doses affect lameness over time. Furthermore, the study used a visual analogue scale for lameness assessment, which, while standardised, may still be subject to observer bias. The reliability of these findings could be improved by using more objective measures such as force plate analysis (Voss et al., 2007).

Owner-reported outcomes were used to monitor the dogs’ progress; however, subjective measures like these are prone to bias and variability in interpretation. Future studies should consider using more objective or standardised measures of health and quality of life, such as clinical measurement instruments (CMIs). For example, the Canine Brief Pain Index (CBPI) or the Liverpool Osteoarthritis in Dogs (LOAD) scale, which assess various domains affected by pain (e.g., quality of life and daily activity levels), could provide more consistent and reliable data (Walton et al., 2013).

Finally, the short duration of the study (6 weeks) limits the ability to assess the long-term effects of laser therapy on NSAID dose, lameness, and pain scores. Future studies with longer follow-up periods are needed to evaluate the lasting effects of laser therapy in combination with NSAIDs.

In conclusion, while the study by Looney et al. (2018) provides promising evidence regarding the use of laser therapy as an adjunct to NSAID treatment for canine OA, the small sample size and some methodological limitations mean the findings are only preliminary. Larger, more robust studies are necessary to definitively determine the efficacy of concurrent laser therapy and NSAID treatment in managing OA in dogs.

## Methodology

**Search Strategy:** 

Databases searched and dates covered:	CAB Abstracts on the OVID interface 1973 – 2024 Week 42 PubMed accessed via the NCBI website 1920 – October 2024
Search strategy:	CAB Abstracts: ((Dog or dogs or bitch* or canine*).mp. Or dogs/ or exp bitches/) and ((Osteoarthritis or OA or osteo-arthritis).mp. Or exp osteoarthritis/) and ((laser* or photobiomodulat* or PBMT or LLLT).mp. Or exp lasers/) and ((non-steroidal* or “non steroidal*” or nonsteroidal* or NSAID* or cyclooxygenase* or carprofen or deracoxib or firocoxib or metacam or galliprant).mp.) PubMed: (Dog or bitch or canine) and (Osteoarthritis or OA or osteo-arthritis) and (laser or photobiomodulation or PBMT or LLLT) and (non-steroidal or “non steroidal” or nonsteroidal or NSAID or cyclooxygenase or carprofen or deracoxib or firocoxib or metacam or galliprant)
Dates searches performed:	23 Oct 2024

**Exclusion / Inclusion Criteria:** 

Exclusion:	Not specifically studying the effect of concurrent laser and NSAID therapy on osteoarthritis. Papers not written in English. Book chapters, conferences, and review papers.
Inclusion:	Controlled clinical trials and pilot studies.

**Search Outcome:** 

Database	Number of results	Excluded – not specific to dogs or did not answer the PICO question	Excluded – book chapters, conferences, review papers, and narrative articles	Excluded – papers not written in English	Total relevant papers
CAB Abstracts	6	1	4	0	1
PubMed	5	3	1	0	1
Total relevant papers when duplicates removed					1

## Acknowledgements

Thank you to Clare Boulton for helping me create the search strategy and running the search. Thank you to Alison Mann and Catherine Dean for the support while writing this Knowledge Summary.

## ORCiD

Ciar Fitzpatrick: https://orcid.org/0009-0000-0356-1362

## Conflict of Interest

The author declares no conflicts of interest.
